# Supplementary material for: Uptake of malaria vaccine (RTS,S/AS01) among children aged 6–24 months: A cross-sectional survey conducted in the Western region of Cameroon, 1 year following the vaccine introduction
Source: PLOS Glob Public Health. 2026 Feb 13;6(2):e0005304. doi: 10.1371/journal.pgph.0005304 (PMC12904457; doi:10.1371/journal.pgph.0005304)
Supplement: Attachment — Submitted filename: Response to reviewers.docx [file pgph.0005304.s003.docx]

Response to reviewers

PGPH-D-25-02818

**Uptake of malaria vaccine (RTS,S/AS01) among children aged 6–24 months: a cross-sectional survey conducted in the Western region of Cameroon, 1 year following the vaccine introduction**

| Comments | Response to comments | Adjustment made from the comments |
| --- | --- | --- |
| **Editor comments** |  |  |
| 1. Please amend your detailed online Financial Disclosure statement. This is published with the article. It must therefore be completed in full sentences and contain the exact wording you wish to be published.  a) State the initials, alongside each funding source, of each author to receive each grant. For example: “This work was supported by the National Institutes of Health (####### to AM; ###### to CJ) and the National Science Foundation (###### to AM).”  b) State what role the funders took in the study. If the funders had no role in your study, please state: “The funders had no role in study design, data collection and analysis, decision to publish, or preparation of the manuscript.” | The instructed amendments have been made online. | Details on the source of funding and the role of funders have been provided as requested. |
| 2. Please ensure that the funders and grant numbers match between the Financial Disclosure field and the Funding Information tab in your submission form. Note that the funders must be provided in the same order in both places as well. | The instructed amendments have been made online. | We ensure that the funders and grant numbers match between the Financial Disclosure field and the Funding Information tab in our submission form |
| 3. Please update your online Competing Interests statement. If you have no competing interests to declare, please state : “The authors have declared that no competing interests exist.” | The instructed amendment have been made online. | Details on Competing interest have been provided as requested. |
| 4. In the online submission form, you indicated that “The database is available and accessible from the corresponding author.”.  All PLOS journals now require all data underlying the findings described in their manuscript to be freely available to other researchers, either  a) In a public repository,  b) Within the manuscript itself, or  c) Uploaded as supplementary information.  This policy applies to all data except where public deposition would breach compliance with the protocol approved by your research ethics board. If your data cannot be made publicly available for ethical or legal reasons (e.g., public availability would compromise patient privacy), please explain your reasons by return email and your exemption request will be escalated to the editor for approval. Your exemption request will be handled independently and will not hold up the peer review process, but will need to be resolved should your manuscript be accepted for publication. One of the Editorial team will then be in touch if there are any issues.  For further assistance, you may go to: <http://journals.plos.org/globalpublichealth/s/data-availability> | Following the editor’s comment, the database has been uploaded as supplementary information. | Following the editor’s comment, the database has been uploaded as supplementary information. |
| 5. Please provide separate main figure files in .tif or .eps format only and ensure that all files are under our size limit of 10MB.  For more information about how to convert your figure files please see our guidelines: <https://journals.plos.org/globalpublichealth/s/figures> | The main figure has been removed following the instruction from comment 7. | Figure 1 has been removed and its citation in the manuscript has been removed as well (Line 95 and Line 98 in the revised manuscript with track changes). |
| 6. We do not publish any copyright or trademark symbols that usually accompany proprietary names, eg (R), (C), or TM (e.g. next to drug or reagent names). Please remove all instances of trademark/copyright symbols throughout the text, including ™ on page 18. | The manuscript has been amended following the instructions given. | We have removed all instances of trademark/copyright symbols throughout the text, including ™ on page 23, line 392 (pages on the actual version in track change). |
| 7. Some material included in your submission may be copyrighted. According to PLOS’s copyright policy, authors who use figures or other material (e.g., graphics, clipart, maps) from another author or copyright holder must demonstrate or obtain permission to publish this material under the Creative Commons Attribution 4.0 International (CC BY 4.0) License used by PLOS journals. Please closely review the details of PLOS’s copyright requirements here: PLOS Licenses and Copyright. If you need to request permissions from a copyright holder, you may use PLOS's Copyright Content Permission form.  Please respond directly to this email or email the journal office and provide any known details concerning your material's license terms and permissions required for reuse, even if you have not yet obtained copyright permissions or are unsure of your material's copyright compatibility.  Potential Copyright Issues:  Figure 1: please (a) provide a direct link to the base layer of the map (i.e., the country or region border shape) and ensure this is also included in the figure legend; and (b) provide a link to the terms of use / license information for the base layer image or shapefile. We cannot publish proprietary or copyrighted maps (e.g. Google Maps, Mapquest) and the terms of use for your map base layer must be compatible with our CC-BY 4.0 license.  Note: if you created the map in a software program like R or ArcGIS, please locate and indicate the source of the basemap shapefile onto which data has been plotted.  If your map was obtained from a copyrighted source please amend the figure so that the base map used is from an openly available source. Alternatively, please provide explicit written permission from the copyright holder granting you the right to publish the material under our CC-BY 4.0 license.  Please note that the following CC BY licenses are compatible with PLOS license: CC BY 4.0, CC BY 2.0 and CC BY 3.0, meanwhile such licenses as CC BY-ND 3.0 and others are not compatible due to additional restrictions.  If you are unsure whether you can use a map or not, please do reach out and we will be able to help you. The following websites are good examples of where you can source open access or public domain maps:  * U.S. Geological Survey (USGS) - All maps are in the public domain. (http://www.usgs.gov)  * PlaniGlobe - All maps are published under a Creative Commons license so please cite “PlaniGlobe, http://www.planiglobe.com, CC BY 2.0” in the image credit after the caption. (http://www.planiglobe.com/?lang=enl)  * Natural Earth - All maps are public domain. (http://www.naturalearthdata.com/about/terms-of-use/) | The boundaries of the different districts were gotten from the database of the ministry of public health in Cameroon. Coordinate points gotten from the households visited during the data collection period were inserted, resulting to the map presented. Given that there is no official procedure to obtain Copyright, we decided to delete the map from the manuscript. | Figure 1 has been removed and its citation in the manuscript has been removed as well (Line 95 and Line 98 in the revised manuscript with track changes). |
| 8. If the reviewer comments include a recommendation to cite specific previously published works, please review and evaluate these publications to determine whether they are relevant and should be cited. There is no requirement to cite these works unless the editor has indicated otherwise. | There was no recommendation from reviewers requesting to cite specific previously published work. | No action from the comment. |
| **Reviewer 1** |  |  |
| 1. « Malaria remains a major public health challenge despite multiple interventions that have reduced its prevalence and impact globally and in Cameroon. The introduction of new malaria vaccines is a welcome addition to the malaria control tool kit. The authors have reported on a survey in which they determined the coverage, completeness and timeliness of the distribution of a malaria vaccine in the western region of Cameroon. They furthermore assed the impact of factors such as knowledge and perception of the malaria vaccine on the studied parameters. They found extremely high drop out rates and negative perceptions of the malaria vaccine. This is surprising since the expanded program on immunization (EPI) vaccines' uptake in Cameroon is generally high.» | We thank the reviewer for his comment. Based on the sampling type used for the recruitment of our study participants (stratified random sampling) and our data sources (children vaccination status collected from children’s EPI vaccination booklet); we are confident that the probability that our estimates are biased has been limited. The observed high dropout rate in our study is consistent with past studies conducted in Cameroon by the National institute of statistics and other research teams. We have however illustrated this by providing references of studies conducted in Cameroon where the dropout rate ranges from 48-49% for other EPI vaccines with the same target:  [https://www.DHS Report](https://www.bing.com/ck/a?!&&p=bcfebfcd3422625f4cceaa2cf0ca040ae91ae92f7ffde437e01f632090e8cd7dJmltdHM9MTc2NDYzMzYwMA&ptn=3&ver=2&hsh=4&fclid=1d2b62fc-581e-68f0-2733-744959ff6984&psq=rapport+de+l%27EDS+2018&u=a1aHR0cDovL29uc3AubWluc2FudGUuY20vc2l0ZXMvZGVmYXVsdC9maWxlcy9wdWJsaWNhdGlvbnMvMjQzL0VEUyUyMDIwMTglMjBJbmRpY2F0ZXVycyUyMGNsJUMzJUE5cy5wZGY)  <https://pubmed.ncbi.nlm.nih.gov/37658309/> | No action has been taken. |
| 2. « The interpretation of their results would have been more convincing if they had a controlled group taking a comparator vaccine, or the same group administered one of the EPI vaccines for comparison. As it stands their study is seriously flawed by the lack of appropriate controls which makes interpretation difficult, if not impossible » | The study was planned to estimate the coverage, timeliness and completeness of the RTS,S/AS01 vaccine. The aim was to propose recommendations to improve the malaria vaccine demand and completeness for the targeted population. We don’t think that there would have been any convincing recommendations if we compared these parameters with those of other vaccines that have different schedules. | No action has been taken. |
| 3. « Furthermore the authors did not attempt to determine the efficacy and impact of the vaccine on the prevalence and morbidity of malaria during the study period. As it stands this looks like a progress report on an interesting and relevant study which which needs to be properly controlled in order to yield credible results. » | We did not plan to estimate the efficacy of the malaria vaccine from this community-based survey as it can’t be done with this type of study design. We attempted to assess the uptake of the vaccine and to explore the need for interventions to improve it (via caregiver’s perception). The plan to assess the real-life effectiveness of the malaria vaccine in our context is in progress. | No action has been taken. |
| 4. « To improve on the manuscript, the authors should include the name of the malaria vaccine studied i.e., RTS, S in the title (which should be shortened) and the Abstract. » | The name of the vaccine has been added to the title of the article (Line 1) and to the abstract (Line 29). | The name of the vaccine has been added to the title of the article (Line 1) and to the abstract (Line 29). |
| **Reviewer 2** |  |  |
| 1.The manuscript requires proof editing for grammar. | The entire manuscript has been carefully proof-edited to improve grammar, clarity and coherence. | Corrections are documented in track change across the manuscript. |
| 2.  Provide clearer definition for "positve perception" and specify how variables were dichotomised | The definition of “positive perception” has been added and the process of dichotomizing variables has been clarified in the Data Analysis section of the manuscript (Line 160-166 in the revised manuscript with track changes). | The definition of “positive perception” has been added and the process of dichotomizing variables has been clarified in the Data Analysis section of the manuscript (Line 160-166 in the revised manuscript with track changes). |
| 3. Explain how weighing was applied in the analysis. | We have expanded the analysis section to describe how sampling weights were applied to account for the cluster sampling design (Line 147-151 in the revised manuscript with track changes). | We have expanded the analysis section to describe how sampling weights were applied to account for the cluster sampling design (Line 147-151 in the revised manuscript with track changes). |
| 4. Clarify operational definitions of timeliness since these are non-standard for malaria vaccines. | The operational definition (The numerator and the denominator of timeliness) is stated in **Table 1** of the manuscript. The timeliness was estimated considering a child is vaccinated within the first month following the recommended age for a child to be vaccinated for the first and second dose, and two months following the recommended age for the third dose. | The operational definitions (The numerator and the denominator of timeliness) is stated in **Table 1** of the manuscript. The timeliness was estimated considering a child is vaccinated within the first month following the recommended age for a child to be vaccinated for the first and second those, and two months following the recommended age for the third dose. |
| 5. The data collection process section should be improved | The data collection process has been improved (Line 129 – 139 in the revised manuscript with track changes). | The data collection process has been improved (Line 129 – 139 in the revised manuscript with track changes). |
